# Effectiveness of physical exercise for people with chronic diseases: the EFIKRONIK study protocol for a hybrid, clinical and implementation randomized trial

**DOI:** 10.1186/s12875-020-01298-4

**Published:** 2020-11-06

**Authors:** María Soledad Arietaleanizbeaskoa, Aintzane Sancho, Iñigo Olazabal, Concepcion Moreno, Erreka Gil, Arturo Garcia-Alvarez, Nere Mendizabal, Ibon de la Fuente, Silvia Dominguez, Susana Pablo, Gonzalo Grandes, on behalf of the EfiKroniK group, Maria Soledad Arietaleanizbeaskoa, Gonzalo Grandes, Erreka Gil-Rey, Susana Pablo, Arturo García, Nere Mendizabal, Ibon de la Fuente, Silvia Domínguez-Martinez

**Affiliations:** 1 Primary Care Group on Health, Prevention and Chronic Diseases, Biocruces Bizkaia Health Research Institute, Plaza de Cruces 12, 48903 Barakaldo, Bizkaia Spain; 2 Medical Oncology Group, Biocruces Bizkaia Health Research Institute, Plaza de Cruces 12, 48903 Barakaldo, Bizkaia Spain; 3 Medical Hematology Group, Biocruces Bizkaia Health Research Institute, Plaza de Cruces 12, 48903 Barakaldo, Bizkaia Spain; 4 Medical Mental Health Group, Biocruces Bizkaia Health Research Institute, Plaza de Cruces 12, 48903 Barakaldo, Bizkaia Spain

**Keywords:** Chronic diseases, Physical activity, Functional capacity, Quality of life, Primary care

## Abstract

**Background:**

Chronic illnesses are the leading cause of morbidity and mortality and threaten the sustainability of healthcare systems worldwide. There is limited evidence in terms of the best modality and intensity of physical activity for improving cardiorespiratory capacity and quality of life in patients with chronic conditions. The objective of the EfiKroniK study is to estimate the common effect of innovative, individualized and supervised physical exercise, on cardiorespiratory functional capacity and quality of life across people with different chronic conditions.

**Methods/design:**

This is a multicentre clinical trial with a type I hybrid effectiveness-implementation design, including 370 patients each with one of four different chronic illnesses: solid cancer, blood cancer, chronic obstructive pulmonary disease or schizophrenia. Patients will be randomly divided into two parallel groups, stratified by illness type. Patients in both groups will receive a standard healthy life prescription (PVS, from the Spanish “*Prescribe Vida Saludable*”) and additionally, the EfiKroniK group will be prescribed a physical exercise programme tailored to each patient in terms of intensity in each session. The primary outcome variables will be cardiorespiratory functional capacity and quality of life. The secondary outcome variables will be signs and symptoms, psychological and social factors and specific laboratory parameters. We will also analyse the dose-response effect of the physical exercise programme. Qualitative variables will describe patients’ perception of the utility and suitability of the EfiKroniK programme, as well as their expectations and satisfaction, identifying barriers to and facilitators of the EfiKroniK implementation process through discussion groups. The study will be carried out on an intention-to-treat basis, comparing changes throughout the 1-year follow-up between groups, adjusting for baseline, by performing mixed-effect analysis of covariance. We will estimate the effect of time on repeated measures in each subject and changes in the EfiKroniK and PVS groups over time.

**Discussion:**

The study will provide the data necessary to allow us to prescribe physical exercise in a similar way to a drug and as a key part of the treatment of chronic illnesses within our healthcare system.

**Trial registration:**

NCT03810755.

**Date and version identifier:** October 9, 2020. Version2.0.

## Background

The prevalence of chronic illnesses is still growing and exceeds 80% among people above 65 years of age, accounting for at least 80% of consultations and 77% of expenditure in the public health system in the Basque Country [1, 2]. In particular, cardiovascular and respiratory diseases, cancer and diabetes, together with mental illnesses account for a high proportion of disease burden worldwide [3, 4]. The burden is due to their improved prognosis and creates a growing need to address health problems resulting from these conditions, their treatment and associated comorbidities [5–7]. The strategies proposed for addressing this threat to the sustainability of the health system include empowering patients in terms of health self-management, promotion of wellbeing and independence.

The promotion of healthy habits in healthcare settings has shown to be effective in the prevention and management of a large number of chronic conditions [8]. Specifically, physical activity has a key role to play in patients with cancer, schizophrenia or chronic obstructive pulmonary disease (COPD). These patients tend to be much less active due to limitations related to their illness [9–12].

The benefits of physical exercise are the result of improvements in cardiorespiratory functional capacity and quality and quantity of muscle mass. Specifically, the disruption of the body’s homeostasis caused by exercise activates anti-inflammatory mechanisms and strengthens the immune system, contributing to the fight against the development of tumours in patients with cancer, countering the chronic inflammatory state prevailing in metabolic syndrome associated with antipsychotic medications in patients with schizophrenia and increasing functional capacity in patients with COPD [10, 13, 14].

Let us imagine that everyone with chronic respiratory diseases, various types of cancer and mental illnesses would benefit from the effect of physical exercise [15–17]. How much might their quality of life and independence improve? To what extent might they experience reductions in symptoms, complications and relapses? Notably, more than half of patients with these conditions do not even receive advice on physical exercise from their doctors [18, 19], despite the fact that several clinical trials have shown good outcomes associated with physical exercise in the treatment of patients with chronic conditions [20].

This gap between knowledge and practice is due, on the one hand, to the fact that we still have limited knowledge regarding the therapeutic effects of exercise; in particular, most current data is based on the current generic guidelines regarding intensity, even though various studies have suggested that during exercise at the same relative intensity (for example, 80% of the maximum heart rate [HR]), the metabolic response varies markedly between individuals [21, 22]. In order to be able to prescribe physical activity as we do drugs, we need to know exactly what dose produces the most effective response with the lowest risk [10]. To this end, we must carry out proper monitoring and use widely accepted methods [23]. Specifically, to ensure safety and the stimulus desired, rather than using HR assessed by direct or indirect methods, it might be more accurate to tailor intensity recommendations based on lactate thresholds [24–26], as this approach is considered the gold standard for the individualised exercise prescriptions [27]. The variation in lactate accumulation allows us to assess the metabolic or energetic response of an individual to a given intensity, and determine metabolic thresholds, that is, specifically, aerobic and anaerobic thresholds. Lactate is a metabolite secreted to obtain energy from glucose and which can be re-used as a source of energy during exercise at moderate intensity. At higher levels of intensity, however, when the energy requirements are greater than the capacity of the body to reuse generated lactate, this metabolite starts to accumulate [28]. Further, lactate accumulation is an indicator of a large number of processes occurring in the body as a result of exercise and which are good for the health.

On the other hand, numerous difficulties are encountered in translating the knowledge available to routine clinical practice [29] in health systems that are more focused on treating the pathological aspects of illnesses than in strengthening the health of individuals with these illnesses. In all sectors, growing importance is being placed on integrating the promotion of physical activity into the treatment of individuals with chronic conditions [9, 18, 30] but it remains unclear how to do this in an effective, widespread and sustainable way.

The clinical intervention assessed in EfiKroniK is physical exercise, which has an impact on the pathophysiological mechanisms underlying the aforementioned chronic conditions and facilitates the adaptive response of individuals to the limitations imposed by their condition, improving health-related quality of life, functional capacity and independence [12, 31, 32]. A review being carried out by Nunan and colleagues will strengthen the quality of the evidence regarding exercise as a medicine and will develop a taxonomy for prescribing physical activity [33]. That said, the data currently available, especially for the advanced stages of illness on which EfiKroniK is to focus, are often based on small observational studies that are vulnerable to potential confounders. Further, we have limited knowledge concerning the best modality and intensity of exercise, as well as the minimum therapeutic dose, and its effect at the individual level, to help us identify which patients might most benefit from exercise prescription [11, 32]. All this information is necessary to be able to prescribe physical exercise in a similar way to a drug [3].

## Clinical objectives

The objectives are:

To estimate the common effect of the innovative EfiKroniK programme of physical exercise, supervised by fitness and health professionals, across people with various different chronic conditions (solid or blood cancer, COPD or schizophrenia), compared to that of a general intervention promoting healthy habits (physical activity, balanced diet and smoking cessation). The effectiveness will be explored in terms of:

- Cardiorespiratory functional capacity estimated using a 400-m walk test
- Quality of life and utility, measured using generic and specific questionnaires for each condition
- Symptoms, and psychological and social well-being.

*All of these variables will be assessed on recruitment and at 3, 6 and 12 months of follow-up.

We will also measure specific blood parameters at baseline and 3 months (adiponectin, C-reactive protein, TNF-α, Il-1, Il-6, brain-derived neurotrophic factor [BDNF]).

To assess the dose-response relationship between exercise performed and the aforementioned response variables, as well as the predictive, moderating and mediating role of each illness, its treatment and patient characteristics, to build a predictive model of the clinical outcomes of the programme based on these clinical variables.

To estimate the associated costs for the EfiKroniK group and the standard healthy lifestyle prescription (hereon PVS, from the Spanish “*Prescribe Vida Saludable*” meaning prescribing a healthy lifestyle) group and calculate incremental cost-effectiveness and cost-utility ratios.

### Implementation objectives

- To describe patients’ perception of the usefulness and appropriateness of the EfiKroniK programme, as well as their expectations and satisfaction.
- To identify barriers to and facilitators of the widespread, sustainable and ongoing implementation of EfiKroniK, as well as the adherence of patients, to guide the design of future strategies for implementing and rolling out this programme which will be assessed in future implementation trials.

## Methodology

### Study design

This will be a multicentre pragmatic open-label type I hybrid implementation-effectiveness trial, in which a total of 370 patients each with one of 4 different chronic conditions, namely, solid cancer (*n* = 100) and blood cancers (*n* = 70), COPD (n = 100) or schizophrenia (n = 100), will be randomised to one of two parallel groups, stratified by illness type: the EfiKroniK group (EG) (tailored exercise supervised by fitness and health professionals) or the reference group (PVS group) [34]. We deliberately opted to study diverse illnesses seeking to demonstrate any common effect of physical exercise across all of them, independent of any additional effects on each condition separately, and for this purpose, this design is much more efficient than separate clinical trials for each illness. Cardiorespiratory functional capacity at 3, 6 and 12 months and quality of life at baseline and at 6 and 12 months will be considered the primary outcome variables, and we will compare changes over time in both groups. Additionally, we will explore the perceptions of participants and professionals in terms of the feasibility of the programme, as well as the barriers and facilitators for adherence and continuity.

### Study population

#### Inclusion criteria

Participants are required to be between 18 and 75 years old and diagnosed with: stage IV solid cancer specifically colon, breast or non-small-cell lung cancer, have an Eastern Cooperative Oncology Group ECOG Performance Status of ≤1, and be on standard first-line chemotherapy; malignant blood cancer and have received an autologous transplant or non-localised lymphoma and be on immune therapy; schizophrenia including first-episode psychosis or other psychotic disorders; or clinically stable (no exacerbation, antibiotic treatment, systemic corticosteroid therapy or hospitalization in the previous 30 days) COPD with a BODE index of 3–7 and a life expectancy > 2 years. In addition, the following are required for patients to be eligible for inclusion: good renal, liver and blood function, with haemoglobin levels > 10 g/dl, a platelet count > 50,000, neutrophil count > 1000, and Karnofsky Performance Status score > 60, and an Eastern Cooperative Oncology Group Performance Status score ≥ 1.

#### Exclusion criteria

Patients are to be excluded if they have brain metastasis, high risk of fracture due to bone metastasis, severe emotional instability, cardiorespiratory compromise or uncontrolled infection; relapse or progression of blood cancer; alterations in communication or significant cognitive impairment that might hinder data collection; bronchiectasis or lung disease other than COPD; other comorbidities that might hinder or prevent them from following the exercise programme; or uncontrolled high blood pressure (systolic > 200 or diastolic > 110 mmHg).

### Recruitment

The Oncology, Haematology and Pulmonology services, primary care doctors and the Mental Health Network of Bizkaia have established an active surveillance system to identify patients with the chronic conditions under study. Doctors will inform patients about the study and invite them to participate. After signing an informed consent form, patients will be referred by the study coordination group to their primary care doctor who will gather baseline data. Before each patient’s group allocation is known, a fitness and health professional will measure the study variables at baseline and provide the standardised healthy lifestyle prescription, encouraging physical activity, a balanced diet and smoking cessation.

### Randomisation

**Fig. 1:**
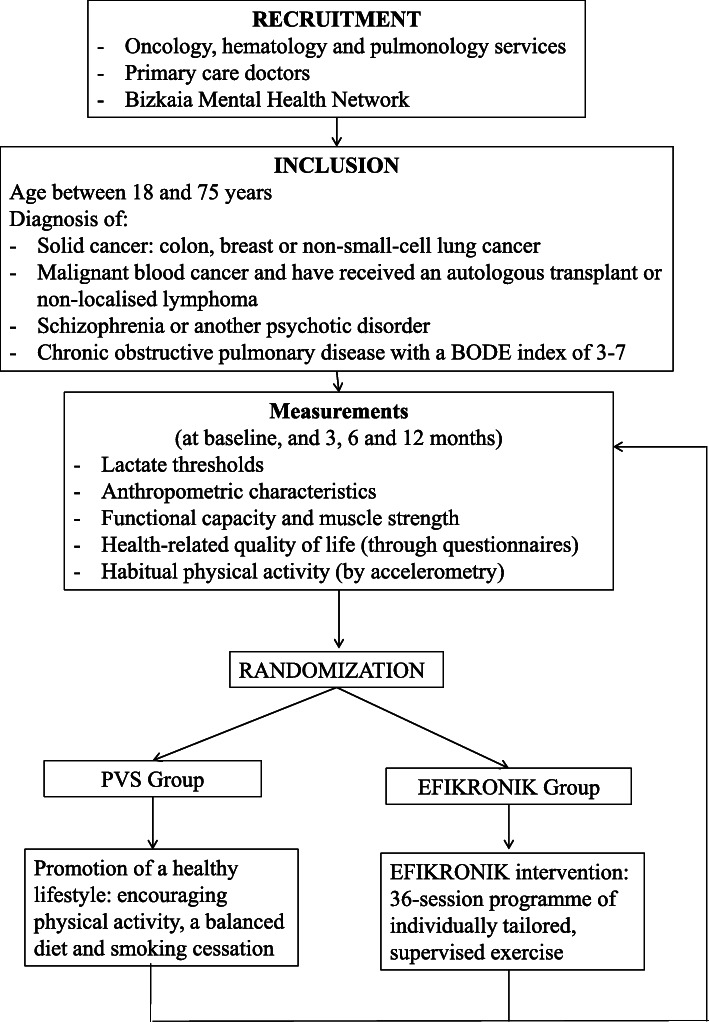
Schematic overview of the process for programme implementation and evaluation

Once informed consent has been obtained and baseline measurements have been taken, patients will be randomised blindly and centrally to either the EG or PVS group, stratified by illness type, at the coordinating centre, the Primary care Research Unit of Bizkaia of the Biocruces Bizkaia Health Research Institute. The randomization will be performed using computer-generated random numbers in a 1:1 ratio, stratified by illness type, with a block size of 4 or 6. (Fig. 1).

### Protocol for the PVS group

On recruitment, all participants will receive a standard healthy lifestyle prescription, encouraging physical activity, a balanced diet and smoking cessation, using the PVS computer tool, which is integrated into the electronic health record. In addition to other measurements at 3, 6 and 12 months, the follow-up will include assessment of potential changes in these habits.

### Protocol for the EfiKroniK group

First phase: the EfiKroniK group are to participate in an innovative physical exercise programme supervised by fitness and health professionals, ensuring patient safety and adjusting the intensity of the exercise to each patient. In this phase, patients are to develop the skills necessary to become an expert regarding the ideal dose of exercise for them. It consists of 36 sessions of exercise of progressively increasing intensity and tailored to the physical condition of each patient, assessed based on metabolic thresholds. Two sessions a week are to be performed in the laboratory, combining aerobic and strength exercises with stretching, under the supervision of fitness and health professionals and one session a week independently near the health centre, monitored with an HR monitor programmed by the fitness and health professionals. In addition to HR measured with the monitor, exercise is monitored with the modified Borg Rating of Perceived Exertion Scale [35] and the appearance of any symptoms.

Second phase: In this phase, patients are to work independently following a physical exercise programme similar to that of the first phase, taking advantage of resources in the community. To make this feasible, patients are to have been trained during the first phase in the type of exercise and appropriate intensity (Borg Scale, HR monitor, symptoms), and at the end of the 3-month training programme, we will contact individuals who can provide support in the community.

We will calculate the real dose of physical exercise each person has been exposed to: cumulative metabolic equivalent (MET)*h/week and time spent doing moderate and/or vigorous intensity of exercise and intensity of exercise under supervision based on the percentage of heart rate reserve (HRR).

#### Intensity of aerobic exercise

The American College of Sports Medicine recommends doing at least 150 min/week of moderate or 75 min/week of vigorous physical activity or a combination of both [36]. Regarding the definition of relative intensity, 45–59% of the HRR corresponds to a moderate intensity. Use of this indicator, however, implies carrying out a maximum stress test, the results of which may be distorted by the high degree of fatigue, weakened immune system and reduced peripheral muscle strength of patients who are receiving intensive treatments and have a low level of fitness [28]. Furthermore, at the same relative intensity (59% of HRR), the metabolic response varies greatly between individuals [21]. For these reasons, we will measure metabolic thresholds in each patient to allow us to: 1) assess patient aerobic capacity and changes therein, given their strong association with maximum rate of oxygen consumption (VO2max), 2) ensure patient safety and provide them with a level of metabolic stimulus that is similar across patients and effective in improving biochemical parameters (e.g., reduction in inflammation and improvements in the immune system, blood glucose levels and lipid profile), and 3) obtain a reference for designing exercise intensity zones for each patient, in terms of speed and HR as well as perceived exertion. In particular, the first lactate threshold (LT1) can be used in this way given the parallel response between the exponential increase in lactate concentrations that occurs after this threshold and the secretion of catecholamines, neurotrophic hormones associated with improvements in cognitive function (BDNF) and anti-inflammatory interleukins (IL-6) involved in reductions in tumour size and reproducibility [37].

The speed at the LT1 determined during an incremental lactate test and the corresponding HR will define the lower limit of the moderate-intensity zone. On the other hand, the speed at the anaerobic threshold, used as a measure of the maximal lactate steady state (MLSS) [36], and the corresponding HR will be taken as the upper limit of the moderate-intensity zone.

Based on the individual metabolic thresholds, we will design five intensity zones. The zone of low-intensity training (LIT) corresponds to exercise carried out at an HR lower than that at LT1 (~ 20% of the HRR) and the zone of moderate intensity to exercise carried out between LT1 and the MLSS (~ 20–85% of HRR or between 1 and 2.3 Mmol/L), this in turn being divided into three equally-sized zones: low-moderate (M1), medium-moderate (M2) and high-moderate (M3) intensity. Finally, the zone of high-intensity training (HIT) (>MLSS, > ~ 85% of the HRR).

During the first month, participants will start carrying out sessions involving endurance exercise, which will be divided into 3 intervals of 8 min each at an M2 intensity, alternating with 2 min at an M1 intensity. In the session in which patients work independently, they will be monitored with HR monitor and it will be explained that they should exercise for 30 min and for as much of that time as possible at M1.

**Fig. 2:**
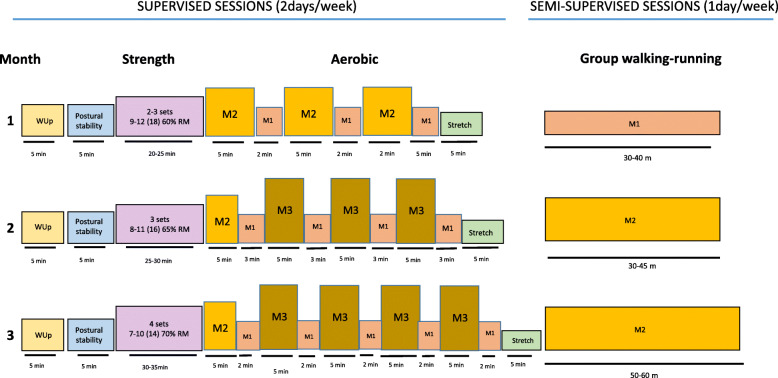
EfiKroniK exercise programme

During the second month, the maximum intensity of the intervals will increase to M3, nearing the anaerobic threshold HR. Specifically, the fully supervised exercise will be divided into four intervals of 5 min at M3, alternating with 3 min at M1, while the indirectly monitored (semi-supervised) session will be of 30 min at M2 (Fig. 2).

Three months after the beginning of the project, the assessments are to be repeated and new intensity zones prescribed, in line with changes in functional capacity as assessed by the lactate tests. At this point, patients are to be given a report of the results of these assessments together with guidelines for exercising independently during the following months. During the same appointment, we will explain to patients how to use the HR zones prescribed during exercise and we will warn them about any potential risk factors detected [37–39]. Given that a relatively high proportion of people on the same training programme do not show a good response in various metabolic parameters [39, 40], patients who adapt well to the training will be recommended to continue exercising at a high-moderate intensity (M3), while those with a poorer response will be encouraged to continue their training at least at low- to medium-moderate intensities (M1-M2).

#### Strength and endurance exercises

Strength training is a key element of the exercise programme in these conditions which are associated with reduced peripheral muscle strength and a low level of fitness. The approach taken consists of exercising the largest muscle groups at high speed and moderate intensity with a moderate load. In each session, 5 exercises are performed involving major muscle groups, namely, the chest, quadriceps, back, hamstring and gluteal muscles, as well as the shoulder, these being performed in 2–3 series of 8–12 repetitions of 16–20 possible exercises (~ 55–65% of the 1 repetition maximum, seeking to avoid excessive muscle damage and inflammation) and using machines, dumbbells, weight bars and plates, elastic straps and wearable weights.

This type of strength training has shown to be effective for releasing anabolic myokines (IL-4, IL-13, IL-15, leukaemia inhibitory factor) and have great therapeutic potential against osteoporosis and sarcopenia/cachexia (responsible for around 20% of cancer-related deaths) associated with metastatic cancer and associated treatments [37, 40], as well as resulting in only mild muscle damage and inflammation.

### Outcome measures

These measurements are made by an interviewer blind to group allocation.

*Primary outcome measure* (baseline, 3, 6 and 12 months):

- Cardiorespiratory functional capacity, as assessed using a 400-m walk test
- Changes in health-related quality of life, as assessed using the 36-item Short-Form Health Survey (SF-36) and disease-specific questionnaires: the European Organization for the Research and Treatment of Cancer Quality of Life Questionnaire (EORTC QLQ-C30) in patients with cancer; and the COPD Assessment Test (CAT) and Chronic Respiratory Disease Questionnaire (CRQ) in patients with COPD.

*Secondary outcome measures* (baseline, 3, 6 and 12 months):

- Muscle strength test measured by the handgrip strength and sit-and-stand tests
- Thresholds for lactate (LT1 and MLSS), HR, and accelerometer activity counts for tailoring the exercises, determined in submaximal walking or running tests. For detecting LT1, a test will be carried out on a 20-m track. This will be an incremental test combining 2-min active period and 1-min rest period. To help the participant adjust their speed in each 2-min period, red markers will be placed every 5 m on the floor and the wall on both sides and two large cones on the floor at each end of the track. Participants will be asked to reach the next red markers each time they hear a beep, continually adjusting their speed but without stopping; that is, they should go faster or more slowly seeking to pass each set of markers at the time of the corresponding beep. The speed will start at 2.4 km/h in the first stage; and will increase by 0.61 km/h each stage [40]. At the end of each stage, the following will be recorded: HR, Rating of Perceived Exertion and lactate concentration, by taking a blood sample from each earlobe to find the minimum lactate equivalent (LEmin) above which the lactate concentration starts to increase exponentially. Hence, the aerobic threshold is defined as the intensity of exercise (speed) at which the lowest recorded lactate level is followed by changes ≥0.1 mmol/L in the next stage and > 0.2 mmol/L the one after [25]. The test will be stopped if the participant becomes exhausted or lactate levels exceed 3 mmol/L.

To identify MLSS, another test will be performed a week after the incremental lactate test to identify the corresponding lactate threshold. Based on certain parameters from this progressive test, detailed below, it has been shown to be possible to estimate the speed at which an individual reaches the MLSS accurately (estimates accounting for 86% of the variance in MLSS with a standard error of the estimate of 0.385 km/h and the difference between the estimated value of MLSS and the true value lying between − 0.77 and 0.81 km/h 95% of the time) [25].

For assessing the MLSS, the patient should return to the health centre where the measurements are taken 1 week later. The infrastructure required is the same as for the incremental lactate test; all that changes is the protocol. An audio recording will be made with two blocks of 10 min separated by a 2-min recovery period, at an MLSS speed estimated using the results obtained in the incremental lactate test and the following equation: MLSS = 3.408 + (1.094·LEmin+ 1 mM) – (0.036·age – (0.013· LEmin HR)) [25].

- Physical activity performed. For this, each participant will be fitted with an Actigraph wGT3X-BT accelerometer on their right hip at the level of the iliac crest and an HR monitor band (Polar OH1). They should wear these monitors for a week and complete a daily record of their activity, to monitor activity counts during different intensities of activity. To analyse these data, we will assess the activity counts, which correlated with LT1 during the incremental test, and thereby be able to assess their weekly physical activity based on personal activity count thresholds.
- Body composition (OMROM body fat percentage), body mass index and abdominal circumference, estimated cardiovascular risk and the onset of any cardiovascular events
- Psychological changes assessed using Goldberg’s 12-item General Health Questionnaire (GHQ-12) and the Duke Social Support Index
- In addition, in the case of schizophrenia, changes in mental state assessed using the Positive and Negative Syndrome Scale (PANSS), and the severity of the patient’s condition and changes therein during the course of the programme assessed using the Clinical Global Impression (CGI) and the Global Assessment of Functioning (GAF) scale
- Lipid profile, as well as levels of C-reactive protein, glucose, insulin and specific exercise-related parameters: adiponectin, BDNF in participants with schizophrenia, TNF-α in those with cancer and Il-1 and Il-6 in those who have COPD or are transplant recipients (at baseline and 3 months).
- Potential predictive, modifying and confounding factors: sex, age, comorbidities (Adjusted Clinical Groups Case-Mix System), risk factors, socioeconomic status, drug treatments and characteristics of each chronic condition.

### Adverse events

Provisional analysis of the data is to be performed by an independent committee to monitor safety. This committee will define indicators in relation to mortality, hospital admissions and cardiac decompensation events, having independent access to the necessary data. Composed of people who are independent of the body in charge of the management of the study and of the researchers and blind to patient group allocation, the committee will review the data every quarter and make recommendations to the team leading the project as to whether the study should be interrupted.

### Follow-up period

One year from the beginning of the intervention.

### Sample size

We expect to recruit a total of 370 patients over 2 years; with a loss to follow-up (including deaths) of 30%, this would provide the study with a power of over 90% to detect differences between the two comparison groups in the primary outcome variable (400-m walk completion time) of at least 6 s after 3 months, 12 s after 6 months and 24 s after 12 months of follow-up as significant (alpha = 0.05), assuming a linear increase in the impact of the exercise over time, a standard deviation of 40 s and intra-patient correlation of 0.6. These differences are less than those found in previous studies.

Regarding the quality of life outcome variable, we would have a power of 90% to detect differences between the two groups of 2.5 points at 3 months, 5 points at 6 months and 10 points at 12 months as significant (alpha = 0.05), for all the dimensions of the SF-36 questionnaire, except for physical and emotional roles (80% power) [40].

### Qualitative evaluation

The knowledge gained from the qualitative evaluation is essential for the designing of a tailored implementation strategy, addressing any organisational and professional barriers that may hinder the adoption of the EfiKroniK programme under routine conditions. This evaluation is to be based on discussion groups exploring the perception of the patients involved in the study to identify barriers that threaten the feasibility of the intervention as well as facilitators of continuation, adherence, adoption, sustainability and suitability of the EfiKroniK programme. We will hold discussion groups of 5 to 8 people to obtain a broad and heterogeneous perspective of the EfiKroniK programme.

These sessions will be audio recorded, transcribed and analysed. Further, the moderator and the observer will take written notes during the fieldwork to complement and triangulate the information recorded. The analysis will be based on theory based and guided by the Consolidated Framework for Implementation Research (CFIR) theoretical implementation framework which comprises 39 constructs classified into 5 domains: intervention characteristics, outer setting, inner setting, characteristics of individuals and process of implementation [41]. We will perform two types of interpretation: inductive and deductive. First, the inductive analysis will follow a thematic analysis by identifying patterns of meaning, based on grounded theory, applying the following strategies: open coding and development of initial categories; “memoing” to capture ideas and reflections of the interviewer and the research team as they evolve over the course of the study; and interaction diagrams and discussion sessions to bring together all the details and help to make sense of the data in relation to the emerging theory and conceptual maps. Secondly, the deductive analysis will consist of the identification of patterns of meaning related to the CFIR constructs, classifying the elements of the discourse generated into the categories of the theoretical framework.

### Statistical analysis

The statistical analysis will be performed on an intention-to-treat basis, comparing changes observed from baseline throughout the year of follow-up in participants assigned the EG and PVS group, adjusting for baseline, using mixed-effect analysis of covariance (linear for continuous outcomes and logistic for dichotomous outcomes). The effect of time on the repeated measures in each participant will be estimated and the hypothesis of significantly different (*p* < 0.05) changes in the EG and PVS group will be tested, assessing a treatment group-time interaction term. These models will include an additional effect characteristic of each group and the interaction between the conditions and the effect of the intervention (GE/PVS). The comparisons will be adjusted for potential confounders. The aforementioned effects will be considered fixed and patients and primary care centres will be included as random effects in the intercept, time slope and effect of the programme. Models will be simplified with backward and forward selection using Likelihood ratio tests (selection criteria, *p* < 0.05). A sensitivity analysis will be performed including/excluding outliers. Based on these models, the effect attributable to the intervention will be estimated, calculating the mean differences between groups estimated at each of the follow-up points (adjusted odds ratios for dichotomous outcome variables) and 95% confidence intervals. Before such analysis, potential interaction effects between the specific illness group of the participant and the effect of the interaction would have been ruled out. Regarding missing values, these longitudinal mixed effect models are one of the best approaches for managing missing data. Analysis will be performed by pre-established groups to test hypotheses that the programme is more effective in patients with mental illness, young people, males, those with a high socioeconomic status, less advanced stages of illness and those with certain comorbidities, evaluating an interaction term between these covariates and the intervention (*p* < 0.005). An analysis will also be carried out by protocol, considering the actual dose of physical activity to which each participant has been exposed. Finally, the cost-effectiveness and incremental cost-utility ratios and their confidence intervals will be estimated using resampling techniques (bootstrapping) and a sensitivity analysis will be performed, changing the assumptions of the analysis. The statistical analysis will be performed with SAS and R packages. No imputation method will be used to handle missing data since longitudinal mixed models based on maximum likelihood estimation are more appropriate to deal with missing data [42] than common imputation methods such as last observation carried forward, complete case analysis or other possible forms of imputation.

### Quality control

Various processes have been undertaken seeking to guarantee the quality of the study data, and thereby maximise the validity and reliability of the measurement of outcomes and study findings. These are:

- Preparation of documents for the study process including fieldwork manuals, material for training concerning intervention measurements, educational leaflets and lists of measurements for exercise and health professionals
- Written documentation: electronic and hard copies of the protocol, signed consent forms, and patient results stored under lock and key
- Training for those in charge of the standardisation of the study process
- Training for exercise and health professionals concerning the study characteristics and procedures, and, in particular, the quality of life interview to be carried out with the patients
- Holding of regular meetings: with the coordinator of the study and with the principal investigator for auditing trial conduct (weekly)
- Quarterly meetings and daily contact by email with the members of the EfiKroniK group and all the participating centres
- Production of monthly progress reports.

### Limitations

The structure of the study makes it impossible to blind the participants or the interveners; however, blind outcome assessors will be used. The monitoring of this intervention and data collection is complex, and therefore, data will be collected using several appropriate quality control processes, and efforts will be made to ensure standardization of the intervention. In addition, to avoid contamination of the control group, interveners will be trained, and a pilot study will also be carried out.

### Ethical and legal aspects

This study protocol complies with the Declaration of Helsinki and its revisions, as well as with good clinical practice. The Ethics Committee of the Basque Country approved the study in the health centres, and the conduct of the study will be monitored to ensure it implemented in compliance with the established regulations. Regarding data confidentiality, only the study researchers have access to the data of individuals who agree to participate in the study, in compliance with the Organic Act 15/1999 of December 13, on the protection of personal data and its 2011 revision.

## Discussion

This study seeks to make a substantial contribution to our knowledge concerning the effectiveness of an exercise programme that is supervised and tailored for patients with chronic illnesses run in primary care under conditions of routine clinical practice. It will investigate the minimal therapeutic dose with which the exercise stimulus received by a patient is sufficient to generate an observable improvement in his/her state of health, as well as the effect of tailoring the dose, which will enable us to identify which patients would benefit most from the prescription of exercise. Additionally, it will explore the common effect of exercise across people with a diverse group of chronic illnesses, independent of any additional effects on each of the conditions, this design being more innovative than the undertaking of separate trials for each illness.

Physical exercise interventions in healthcare settings for the treatment of chronic illnesses are still scarce [18]. EfiKroniK is a physical exercise programme carried out in a real healthcare setting in primary care exercise laboratories supervised by professionals trained in the optimisation of results obtained from exercise in terms of its therapeutic effects and impact on patient quality of life. Moreover, the exercise prescription is tailored to the status of patients at the time. This experience will provide an example of a study run from primary care that is well coordinated with hospital oncology, haematology and pulmonology departments and the local mental health network.

Physical exercise has been shown to act as a medicine in numerous chronic diseases, with a beneficial effect on their pathogenesis and symptoms [10]. Nonetheless, we still do not know the minimum dose necessary to obtain such benefits. The EfiKroniK programme will study the minimum therapeutic dose through the prescription of individualized physical exercise based on the metabolic response of each patient to a specific stimulus and the stage of their illness. We will also take into account the current recommendations to combine moderate-intensity aerobic exercises with muscle strength training [35].

Adherence to an active lifestyle is particularly important in patients with chronic illnesses as, given the characteristics of their condition, they tend to be more sedentary [43–45]. In EfiKroniK, we seek to confirm whether participating in an intervention of this sort would help to change physical activity habits and thereby increase the long-term benefits. Further, based on these data and those concerning adherence to the programme, we will perform cost-effectiveness and cost-utility analysis to confirm the feasibility of a programme such as this in healthcare services.

At the same time, the knowledge gained through the qualitative evaluation will be key the designing of a tailored implementation strategy to overcome the organizational and professional barriers that may hinder the adoption of the EfiKroniK programme under routine conditions which will be assessed in future implementation trials [44, 45]. All these data are necessary to make it possible to prescribe physical exercise in a similar way to a drug [33].

## Data Availability

Data sharing is not applicable to this article as no datasets were generated or analysed for preparation of the study protocol.

## Abbreviations

BDNF: Brain-derived neurotrophic factor
COPD: Chronic obstructive pulmonary disease
EG: EfiKroniK group
HR: Heart rate
HRR: Heart rate reserve
LT1: The first lactate threshold
MET: Metabolic equivalent
MLSS: Maximal lactate steady state
PVS: from the Spanish “*Prescribe Vida Saludable*”
VO2max: Maximum rate of oxygen consumption
